# 14q32-encoded microRNAs mediate an oligometastatic phenotype

**DOI:** 10.18632/oncotarget.2920

**Published:** 2015-02-18

**Authors:** Abhineet Uppal, Sean C. Wightman, Stephen Mallon, Go Oshima, Sean P. Pitroda, Qingbei Zhang, Xiaona Huang, Thomas E. Darga, Lei Huang, Jorge Andrade, Huiping Liu, Mark K. Ferguson, Geoffrey L. Greene, Mitchell C. Posner, Samuel Hellman, Nikolai N. Khodarev, Ralph R. Weichselbaum

**Affiliations:** ^1^ Department of Surgery, The University of Chicago, Chicago, IL 60637, USA; ^2^ Department of Radiation and Cellular Oncology, The University of Chicago, Chicago, IL 60637, USA; ^3^ Ludwig Center for Metastasis Research, The University of Chicago, Chicago, IL 60637, USA; ^4^ Department of Pathology, Committee on Cancer Biology, The University of Chicago, Chicago, IL 60637, USA; ^5^ Center for Research Informatics, The University of Chicago, Chicago, IL 60637, USA; ^6^ Department of Pathology, Case Comprehensive Cancer Center, Case Western Reserve University, Cleveland, OH 44106, USA; ^7^ The Ben May Department for Cancer Research, The University of Chicago, Chicago, IL 60637, USA

**Keywords:** metastasis, oligometastasis, microRNA, gene expression, gene regulation

## Abstract

Oligometastasis is a clinically distinct subset of metastasis characterized by a limited number of metastases potentially curable with localized therapies. We analyzed pathways targeted by microRNAs over-expressed in clinical oligometastasis samples and identified suppression of cellular adhesion, invasion, and motility pathways in association with the oligometastatic phenotype. We identified miR-127-5p, miR-544a, and miR-655-3p encoded in the 14q32 microRNA cluster as co-regulators of multiple metastatic pathways through repression of shared target genes. These microRNAs suppressed cellular adhesion and invasion and inhibited metastasis development in an animal model of breast cancer lung colonization. Target genes, including *TGFBR2* and *ROCK2*, were key mediators of these effects. Understanding the role of microRNAs expressed in oligometastases may lead to improved identification of and interventions for patients with curable metastatic disease, as well as an improved understanding of the molecular basis of this unique clinical entity.

## INTRODUCTION

Metastases are the leading cause of cancer-related death. The presence of distant metastases in most adult solid tumors has been synonymous with a fatal outcome. However, it is increasingly recognized that distant metastases may not always be numerous and widespread. We proposed that during the evolution of some tumors, an intermediate state exists between localized cancer and widespread metastatic disease, termed oligometastasis, which is characterized by a limited extent of metastatic disease [1–3]. The clinical significance of oligometastasis is that some patients with limited metastatic disease are curable with metastasis-directed therapies, such as surgery, stereotactic radiotherapy, or tumor ablation, whereas most patients with metastatic disease are incurable even with systemic therapies [3].

Nevertheless, little is known regarding the molecular basis of oligometastasis, especially the differences between oligometastasis and widespread metastatic dissemination (i.e. polymetastasis). Recent studies have demonstrated differential patterns of microRNA expression in oligometastatic patients as compared to polymetastatic patients [4, 5]. MicroRNAs are small, non-coding RNA molecules which function in RNA silencing and post-transcriptional regulation of gene expression [6]. The role of microRNAs in the regulation of metastases has been increasingly recognized, and individual microRNAs have been implicated both in oncogenic and tumor-suppressor pathways involved in multiple steps of the metastatic cascade [7–15]. A common feature of microRNA-dependent gene regulation is that several microRNAs can regulate a single target gene while an individual microRNA can broadly regulate hundreds to thousands of different genes. These findings raise the possibility that specific microRNAs may play a key role in the regulation of an oligometastatic phenotype.

In the current report, we identified four microRNAs encoded in the 14q32 locus (miR-127-5p, miR-369-3p, miR-544a, and miR-655-3p) and associated with an oligometastatic phenotype in clinical metastasis samples. These microRNAs were over-expressed in metastases from patients with limited metastatic disease, some of whom were cured with local therapy, as compared to those who subsequently developed widespread disease [4, 5]. In experimental models of metastasis, we determined that either ectopic expression of 14q32-encoded microRNAs or stable suppression of targeted genes reduced cell-autonomous metastatic properties *in vitro* and inhibited lung metastasis development *in vivo*. Characterization of molecular pathways regulated by these microRNAs demonstrated co-repression of cytoskeletal organization, cell motility, and TGF-beta signaling pathways primarily through direct inhibition of target genes, including TGFBR2, ROCK2, CDH11 and ICK. Taken together, these findings support a critical role for 14q32 microRNAs in the maintenance of an oligometastatic phenotype through coordinated gene regulation of complex networks determining metastatic potential.

## RESULTS

### Oligometastasis-associated microRNAs target key metastatic pathways

**Figure 1:**
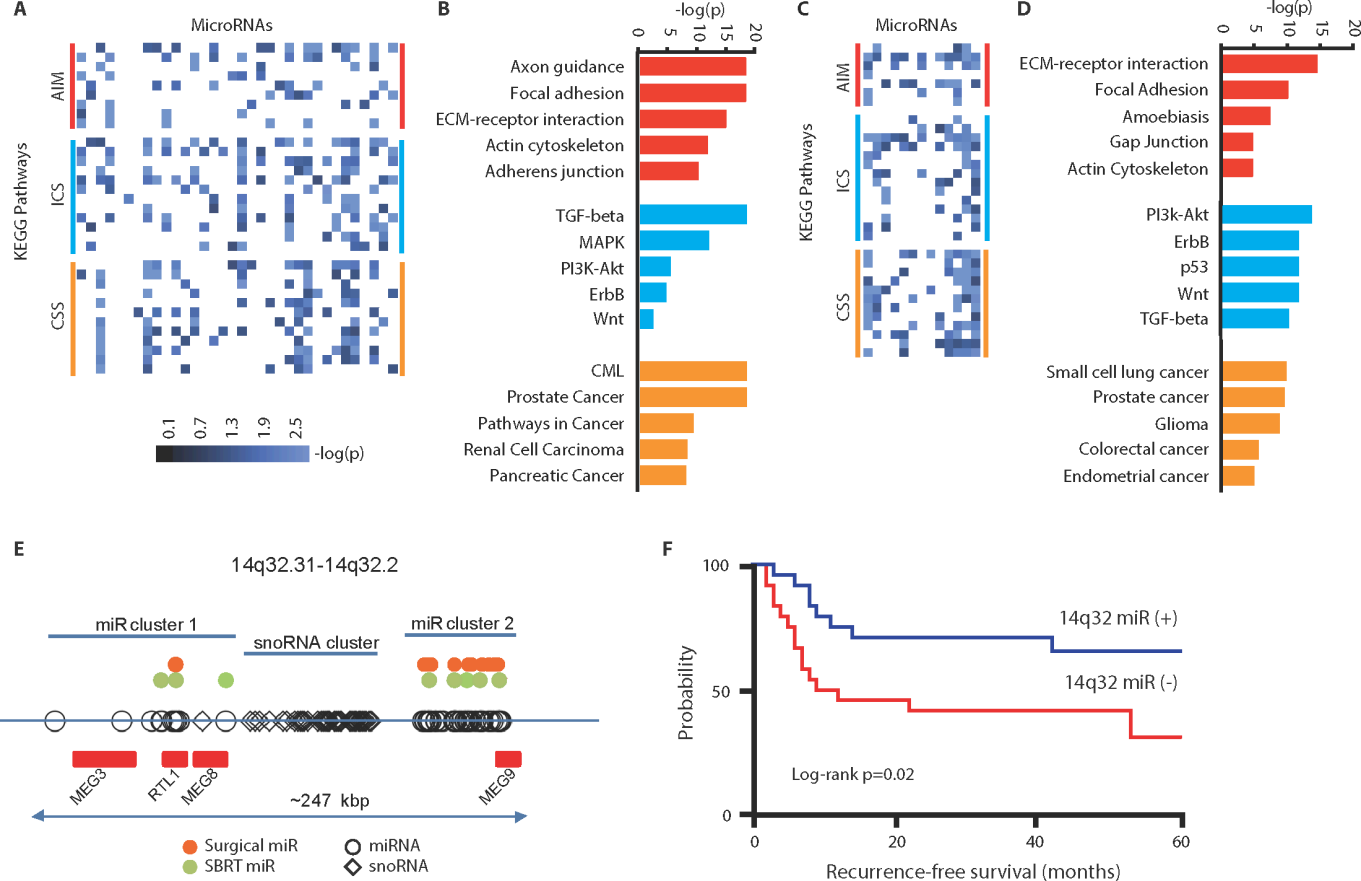
Predicted pathways targeted by oligometastasis-associated microRNAs **(A)** Heatmap diagram demonstrating one-way unsupervised hierarchical clustering of 35 microRNAs up-regulated in surgically resected lung oligometastases as compared to lung polymetastases (surgical dataset) and predicted KEGG target pathways grouped by functions (AIM: adhesion/invasion/motility; ICS: intracellular signaling; CSS: cancer-specific signaling). Values represents −log_10_ of *p*-value where higher values correspond to lower p. **(B)** Most significant predicted microRNA-regulated pathways comprising each functional group of the surgical dataset. Red, blue, and orange colors denote AIM, ICS, and CSS functional groups. **(C)** Heatmap diagram depicting 13 microRNAs over-expressed in oligometastases treated by SBRT and predicted KEGG target pathways. **(D)** Most significant predicted miRNA-regulated pathways from the SBRT dataset. **(E)** A disproportionate number of differentially expressed microRNAs in both datasets are located in the 14q32 genomic locus comprised of two microRNA clusters and one snoRNA cluster (red rectangles are protein-coding genes within locus). **(F)** Over-expression of 14q32-encoded microRNAs (miR-127-5p, miR-369-3p, miR-544a and miR-655-3p) is associated with improved recurrence-free survival in patients undergoing surgical resection of lung metastases (blue: top 50% by expression; red: bottom 50% by expression; *n* = 24 per group).

We sought to characterize the potential roles for oligometastasis-associated microRNAs (miRs) in the regulation of metastatic potential. We first identified predicted gene targets of miRs over-expressed in patient-derived samples of surgically resected lung oligometastases (surgical dataset; [5]) and stereotactic body radiotherapy-treated (SBRT dataset, [4]) oligometastases from various organs. To our knowledge, these data represent the only published miR expression datasets derived from clinically oligometastatic patients. We found a total of 15,921 predicted gene targets for the 35 surgical dataset miRs and 3,451 predicted gene targets for the 13 SBRT dataset miRs (Supplemental Table S1). These gene targets belonged to 49 (surgical) and 52 (SBRT) respective KEGG pathways (Figures 1A–1D). Among these KEGG pathways, we detected three significantly enriched functional motifs common to both datasets (Supplemental Tables S2 and S3). The first functional motif involved the regulation of cell adhesion, cell-extracellular matrix interactions, and cell motility (AIM Group). The second functional cluster consisted of intracellular signaling pathways (ICS Group), including TGF-beta, Wnt, ErbB, PI3K/Akt, and mTOR pathways, all of which are known to mediate metastasis [11, 12, 15]. The third functional motif was broadly represented by cancer-specific signaling pathways (CSS Group). Taken together, these results suggested that miRs associated with an oligometastatic phenotype target genes which mediate important metastatic pathways.

We also determined that several oligometastasis-associated miRs were encoded in a large miR cluster within the DLK1-DIO3 imprinted locus at 14q32.31–14q32.2. Notably, multiple miRs within this locus have been implicated in the repression of epithelial-to-mesenchymal transition (EMT) in tumor cells [8, 16, 17]. Among the 39 differentially expressed miRs in the surgical dataset, 14 (36%) were located in the 14q32 locus, while in the SBRT dataset, 5 of 29 (17%) differentially expressed miRs were present in this locus (Figure 1E). The probabilities of observing these frequencies by chance, as determined using random permutation tests, demonstrated a significant enrichment of 14q32-encoded miRs within oligometastatic samples in both datasets (surgical: *p* < 0.0001; SBRT: *p* = 0.042). These data supported a role for 14q32 miRs in mediating the oligometastatic phenotype.

We focused our analysis on the surgical lung metastasis dataset as it was homogenous with respect to metastatic site, and unlike the SBRT dataset the patients had not been heavily pretreated with other therapies. Candidate miRs were identified by ranking the fourteen 14q32-encoded miRs by the number and significance of AIM targeted pathways (Supplemental Table S2). We prioritized our analysis to AIM-dependent phenotypes given the general importance of interactions between metastatic tumor cells and their microenvironment in metastasis development. We examined four miRs in the 14q32 locus (miR-127-5p, miR-369-3p, miR-544a and miR-655-3p) whose expression was significantly up-regulated in oligometastases as compared to polymetastases and whose target genes were predicted to regulate AIM-related functions. Over-expression of these four miRs was associated with a prolonged recurrence-free interval after surgical resection of lung metastases (Figure 1F). Notably, 60% of patients with elevated expression of these miRs showed no metastatic recurrence at five years of follow-up (vs. 30% in low expressors). In the context of these findings, we tested the hypothesis that these four miRs mediate an oligometastatic phenotype through coordinated repression of pro-metastatic pathways (Supplemental Figure S1).

### 14q32-encoded microRNAs co-regulate target gene expression

**Figure 2:**
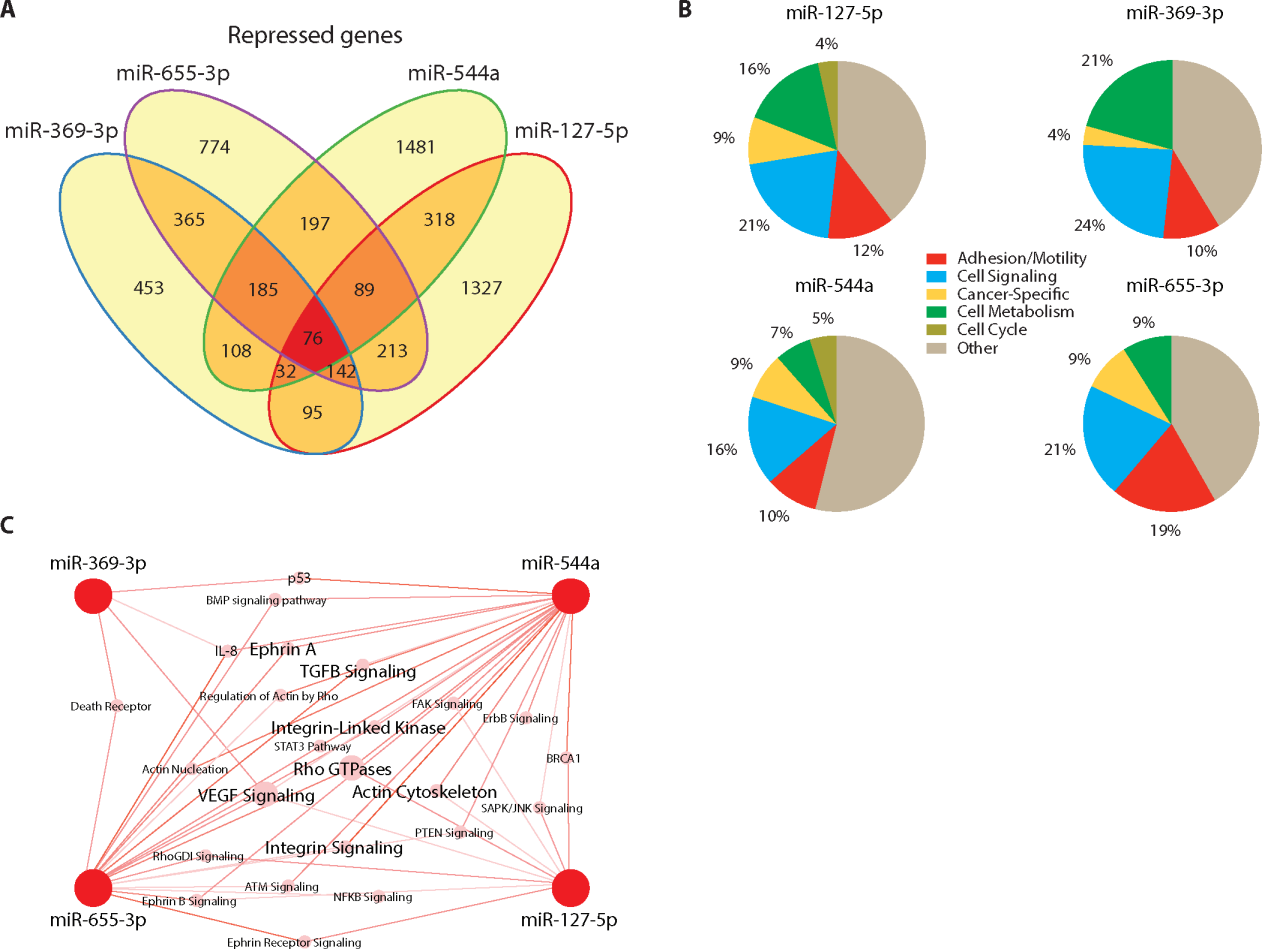
14q32-encoded microRNAs co-regulate target pathways **(A)** Venn diagram illustrating the number of overlapping genes suppressed 1.4-fold or greater (FDR ≤ 5%) after ectopic expression of each microRNA in the metastatic breast cancer cell line MDA-MB-231. **(B)** Functional clusters altered by individual microRNAs. **(C)** Network analysis of 28 pathways related to adhesion/invasion/motility and intracellular signaling altered by two or more miRNAs.

We ectopically expressed miR-127-5p, miR-369-3p, miR-544a, and miR-655-3p in the metastatic breast cancer cell line MDA-MB-231 and performed gene expression profiling to validate the predicted gene and pathway targets. The number of repressed gene probesets as a result of miR over-expression ranged from 1,376 for miR-369-3p to 2,134 for miR-544a. There was a significant degree of overlap in suppressed target genes among the four miRs. In particular, 31% of all probesets were suppressed by two or more miRs, and all four miRs suppressed 76 genes in common (Figure 2A). We found a significant enrichment in the number of co-repressed probesets between combinations of two, three or four miRs, greater than would be expected by chance (*p* < 0.0001 for all pairwise comparisons, Chi-squared test; Supplemental Table S4). These data indicated that miR-127-5p, miR-369-3p, miR-544a, and miR-655-3p miRs co-regulated target gene expression.

Using Ingenuity® Pathway Analysis (IPA) we identified cellular pathways corresponding to repressed target genes and found 47 pathways for miR-127-5p, 23 pathways for miR-369-3p, 146 pathways for miR-544a and 61 pathways for miR-655. These pathways predominantly belonged to overlapping functional groups, including those previously identified in patient-derived metastases (Figure 2B). In particular, pathways belonging to the AIM and ICS groups comprised 40% of all miR-655 suppressed pathways. We found similar results for miR-369-3p (34%), miR-127-5p (33%), and miR-544a (26%). Further analysis demonstrated co-suppression of 28 AIM-related pathways by two or more miRs, including TGF-beta, actin cytoskeleton, FAK, and integrin-linked kinase signaling pathways (Figure 2C). Collectively, these data confirmed co-repression of several metastatic signaling pathways and networks by multiple miRs within the 14q32 locus.

### 14q32-encoded miRs bind 3′ untranslated regions of shared target genes

We hypothesized that the existence of multiple miR binding sites in the 3′ untranslated regions (UTR) of target genes could provide a molecular basis for the observed regulatory networks between these miRs and their target genes. Using miRNA-mRNA binding prediction algorithms, we identified several candidate genes from our microarray analysis which share 3′ UTR miR binding sites, including *TGFBR2*, *ROCK2*, *ICK*, and *CDH11*. Each of these genes has previously been reported to mediate AIM-related pathways involved in tumor metastasis [18–23].

**Figure 3:**
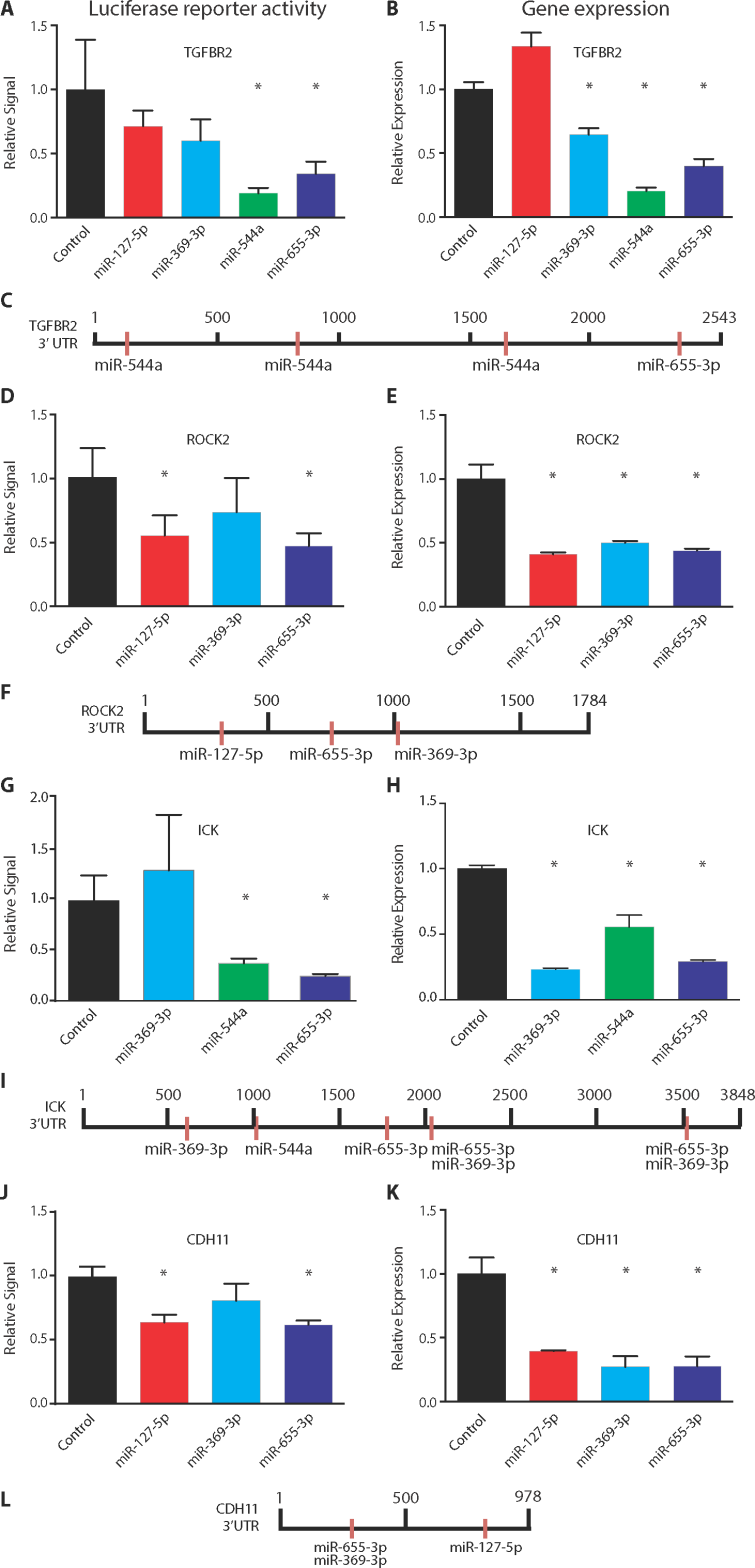
Co-repression of target genes by 14q32-encoded miRNAs Left column **(A, D, G, J)**: luciferase reporter activity assays of HEK 293T cells co-transfected with a luciferase vector containing the 3′ untranslated region (UTR) of the specified gene and each of the tested miRNAs. Right column **(B, E, H, K)**: microarray gene expression values of MDA-MB-231 cells transfected with miR-127-5p, miR-369-3p, miR-544a, miR-655-3p or a non-targeting control. Schematics **(C, F, I, L)** depict the distribution of binding sites of tested miRNAs in the 3′ UTR of tested genes. **(A–C)** TGFBR2. **(D–F)** ROCK2. **(G–I)** ICK. **(J–L)** CDH11. Data represent mean ± SD (*n* = 5 per group). *p ≤ 0.05 compared to control.

We examined post-transcriptional gene regulation using 3′ UTR luciferase reporter assays and quantified the inhibitory effect of each miR on candidate target genes. As predicted, we found direct suppression of TGFBR2 by miR-544a (5.3-fold, *p* = 0.01, Student's *t* test) and miR-655-3p (2.9-fold, *p* = 0.02) (Figure 3A). As a negative control, miR-127-5p failed to significantly decrease luciferase reporter activity (Figures 3A and 3C). Notably, mutagenesis of the predicted TGFBR2 3′ UTR binding sites completely abrogated gene repression by both miR-544a and miR-655-3p (Supplemental Figure S2). We obtained similar results for suppression of ROCK2 by miR-127-5p (1.8-fold, *p* = 0.02) and miR-655-3p (2.2-fold, *p* = 0.01) (Figures 3D and 3F), ICK by miR-544a (2.6-fold, *p* = 0.01) and miR-655-3p (4.0-fold, *p* = 0.01) (Figures 3G and 3I), and CDH11 by miR-127-5p (1.6-fold, *p* = 0.003) and miR-655-3p (1.6-fold, *p* = 0.001) (Figures 3J and 3L). Interestingly, while miR-369-3p suppressed the expression of all candidate genes, it failed to significantly decrease the 3′ UTR luciferase activity despite the presence of miR-369-3p binding sites for *ROCK2*, *ICK*, and *CDH11*. These results suggested the possibility of alternative mechanisms of post-transcriptional regulation by miR-369-3p and/or competition between miR-655-3p and miR-369-3p for shared binding sites. Importantly, the inhibitory effect of each miR on luciferase reporter activity paralleled changes in gene expression after ectopic expression of each miR (Figures 3B, 3E, 3H, and 3K). In addition, the number of binding sites for each miR correlated with the magnitude of post-transcriptional inhibition. Taken together, these data demonstrated that 14q32-encoded miRs negatively regulate 3′ UTRs of multiple pro-metastatic genes and that a given 3′ UTR of a particular gene could be targeted by multiple 14q32 miRs.

### Oligometastatic microRNAs inhibit cell-autonomous metastatic properties of tumor cells

**Figure 4:**
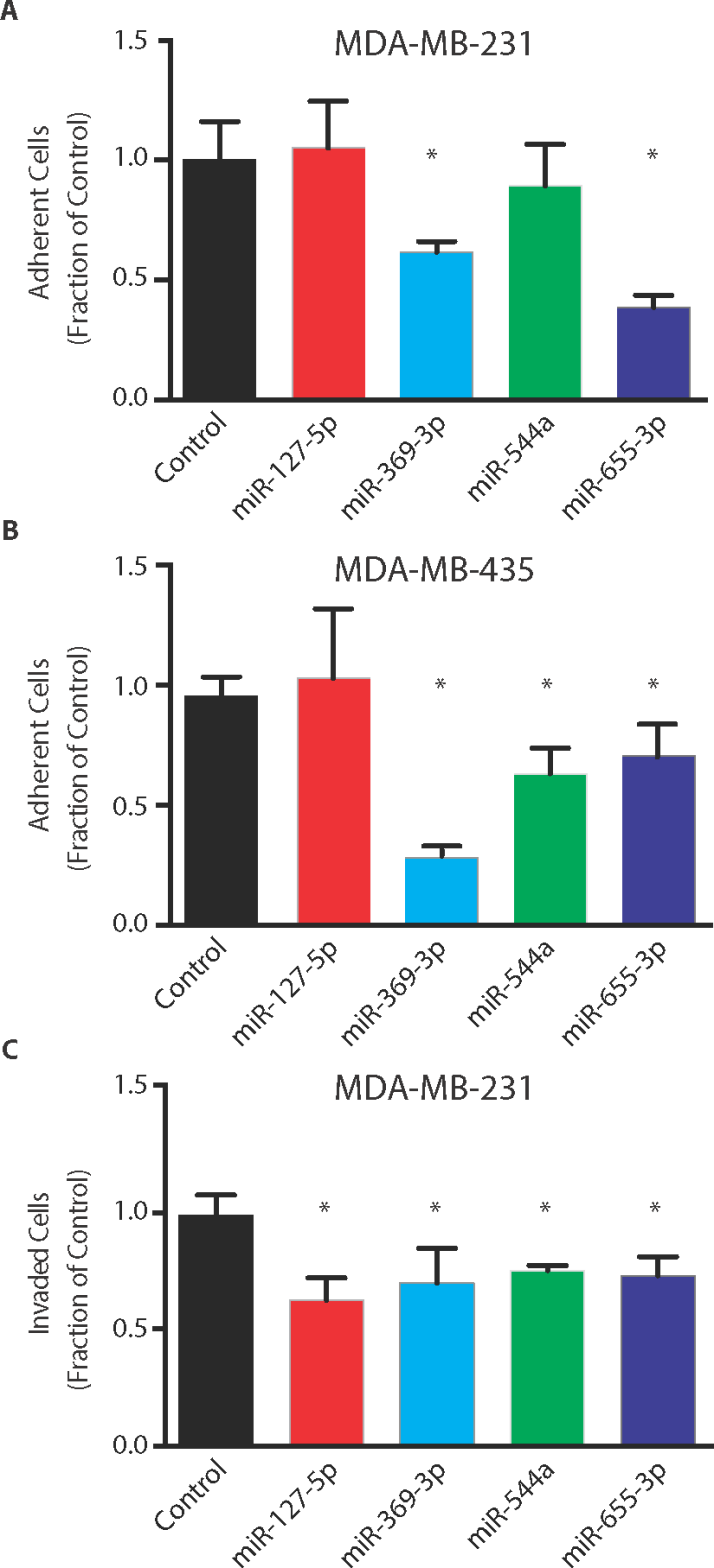
Oligometastatic microRNAs suppress *in vitro* metastatic phenotype Adhesion of MDA-MB-231 **(A)** or MDA-MB-435 **(B)** cells to Matrigel after transient transfection of microRNAs (*n* = 5 per group). **(C)** MDA-MB-231 invasion through Matrigel after ectopic expression of microRNAs (*n* = 3 per group). Data represent mean ± SD. *p ≤ 0.05 compared to control.

To the extent that particular 14q32 miRs co-repressed AIM-related pathways, we examined whether these miRs negatively regulated cellular adhesion and invasion phenotypes. To this end, we performed *in vitro* assays of adhesion and invasion using metastatic cell lines transfected with miR-127-5p, miR-369-3p, miR-544a, miR-655-3p or a non-targeting control (NT). Ectopic expression of either miR-369-3p and miR-655-3p in MDA-MB-231 cells resulted in a significant decrease in adhesion to Matrigel as compared to NT-transfected cells (mean ± std. dev.; miR-369-3p: 61 ± 4%, *p* = 0.007; miR-655-3p: 39 ± 5%, *p* < 0.001; Student's *t*-test, Figure 4A), while ectopic expression of miR-369-3p, miR-544a, and miR-655-3p significantly reduced cellular adhesion of MDA-MB-435 cells (miR-369-3p: 28 ± 1.1%, *p* = 0.0006; miR-544a: 63 ± 11%, *p* = 0.005; miR-655-3p: 71 ± 15%, *p* = 0.05; Figure 4B). All four miRs significantly inhibited the invasion of MDA-MB-231 tumor cells through Matrigel (miR-127-5p: 62 ± 15%, *p* = 0.001; miR-369-3p: 72 ± 20%, *p* = 0.02; miR-544a: 75 ± 10%, *p* = 0.03; miR-655-3p: 73 ± 15%, *p* = 0.01, Figure 4C). These data demonstrated cell-autonomous mechanisms of adhesion and invasion through which particular 14q32-encoded miRs may suppress the metastatic phenotype.

### Oligometastatic microRNAs suppress metastatic lung colonization

**Figure 5:**
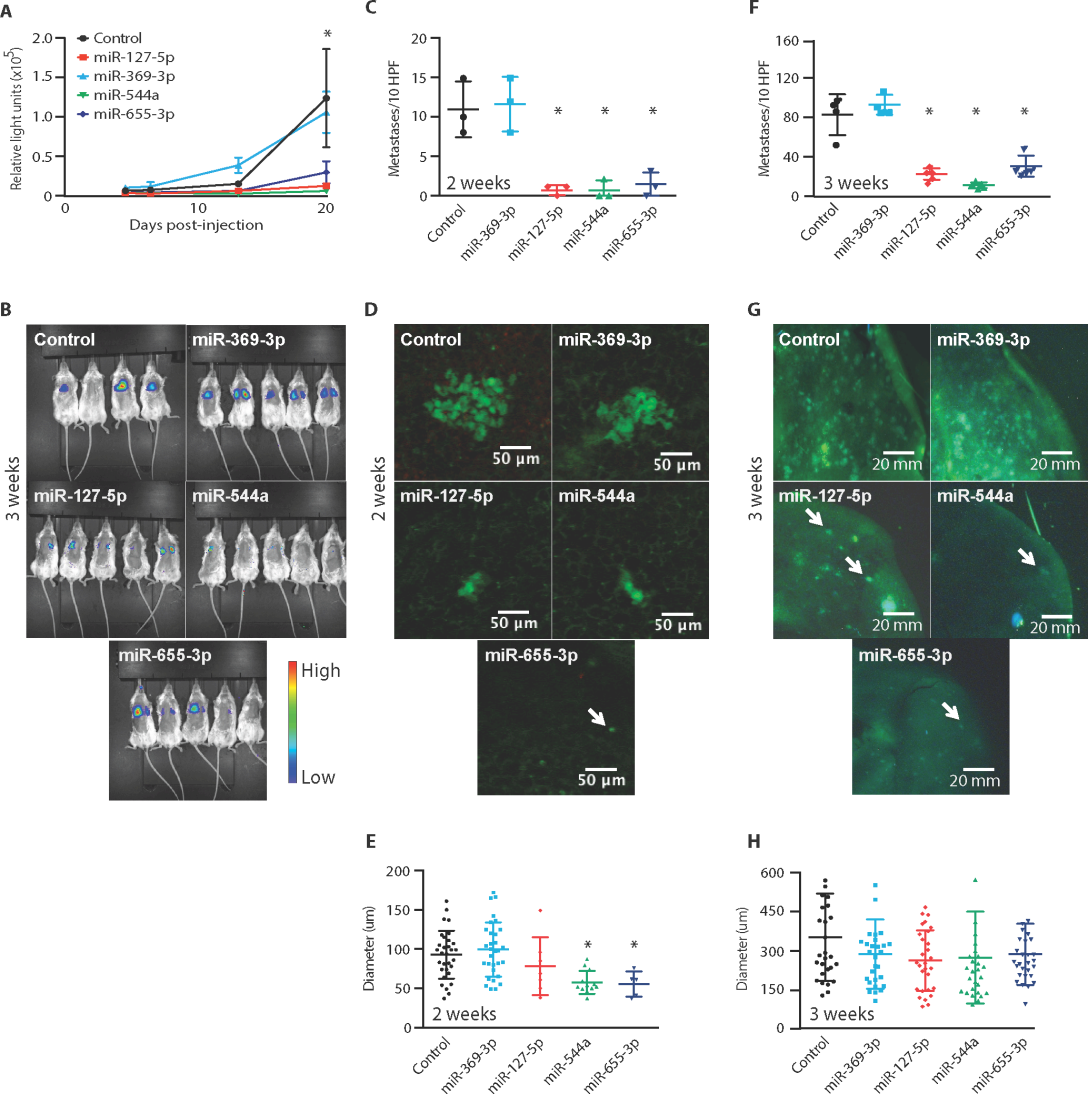
Ectopic expression of 14q32-encoded miRNAs limits lung colonization of metastatic tumor cells **(A)** MDA-MB-231 polymetastatic breast cancer cells co-labeled with luciferase and GFP and transfected with miR-127-5p, miR-369-3p, miR-544a, miR-655-3p or a non-targeting control were injected into the tail veins of NOD/SCID mice. **(B)** *In vivo* whole-animal luciferase imaging at 3 weeks (*n* = 4–5 per group). **(C)** Number of metastases per 10 high-powered fields (HPFs) based on fluorescence imaging of GFP at 2 weeks post-injection demonstrating significantly fewer lung metastases after transfection with miR-127-5p, miR-544a and miR-655-3p (*n* = 3 per group). **(D)** *Ex vivo* photomicrographs of lung colonies at 2 weeks. **(E)** Micro-colony diameter at 2 weeks showing a significant growth delay in miR-544a and miR-655-3p transfected cells. **(F)** Number of metastases per 10 HPFs at 3 weeks post-injection demonstrating significantly reduced lung metastases after transfection with miR-127-5p, miR-544a or miR-655-3p (*n* = 6–8 per group). **(G)** *Ex vivo* photomicrographs of lungs at 3 weeks. Arrows denote individual metastatic colonies. **(H)** Micro-colony diameter at 3 weeks showed no significant differences in size among groups. Data represent mean ± SD. *p ≤ 0.05 compared to control.

We investigated the effect of these 14q32 miRs in metastatically competent tumor cells using an experimental model of metastasis. We utilized the polymetastatic MDA-MB-231 breast cancer cell line co-labeled with green fluorescent protein (GFP) and luciferase [23] and transfected with miR mimics to evaluate the influence of each miR on subsequent metastasis development. We specifically evaluated changes in metastatic potential, as opposed to metastasis initiation, by quantifying lung colonization of metastatic tumor cells injected into the tail veins of NOD/SCID mice. At 2 weeks following injection, we found that miR-127-5p, miR-544a, and miR-655-3p significantly decreased (2.3- to 7.1-fold, *p* < 0.0002, Student's *t*-Test) the luminescence signal in the lungs of mice as compared to control treated animals (Figure 5A). In contrast, miR-369-3p failed to suppress the luminescence signal. Notably, at 3 weeks following injection, we detected even greater reductions in luminescence in miR-127-5p, miR-544a, and miR-655-3p treated groups (3.7- to 12.5-fold, *p* < 0.03, Figures 5A and 5B).

To further characterize lung colonization proficiency, we assessed the GFP signal of *ex vivo* lung tissue using confocal microscopy. At 2 weeks, we identified fewer tumor micro-colonies in the miR-127-5p, miR-544a and miR-655-3p treated groups as compared to controls (mean ± std. dev.; control: 11 ± 3.6; miR-127-5p: 0.6 ± 0.5, *p* = 0.0002; miR-544a: 0.7 ± 1.2, *p* = 0.0001; miR-655-3p: 1.3 ± 1.5; *p* = 0.0007, Student's *t*-test; Figure 5C). In addition, tumor micro-colonies in the miR-544a and miR-655-3p groups were significantly smaller in size (Figures 5D and 5E). At 3 weeks following injection, we found similar differences with respect to micro-colony numbers (control: 78.5 ± 19.5; miR-127-5p: 21.6 ± 5.5, *p* = 0.0004; miR-544a: 10.9 ± 2.9, *p* = 0.0001; miR-655-3p: 29.2 ± 10.1, *p* = 0.0017; Figure 5F); however, there were no differences in micro-colony size between control and miR treated groups (Figures 5G and 5H). It is notable that miR-127-5p, miR-544a, and miR-655-3p each produced a similar phenotype of limited metastatic potential and that no individual miR completely abrogated lung colonization. Together these data demonstrated that miR-127-5p, miR-544a and miR-655-3p suppressed the development of lung metastases through the inhibition of lung colonization.

### Suppression of microRNA target genes phenocopies oligometastatic state

**Figure 6:**
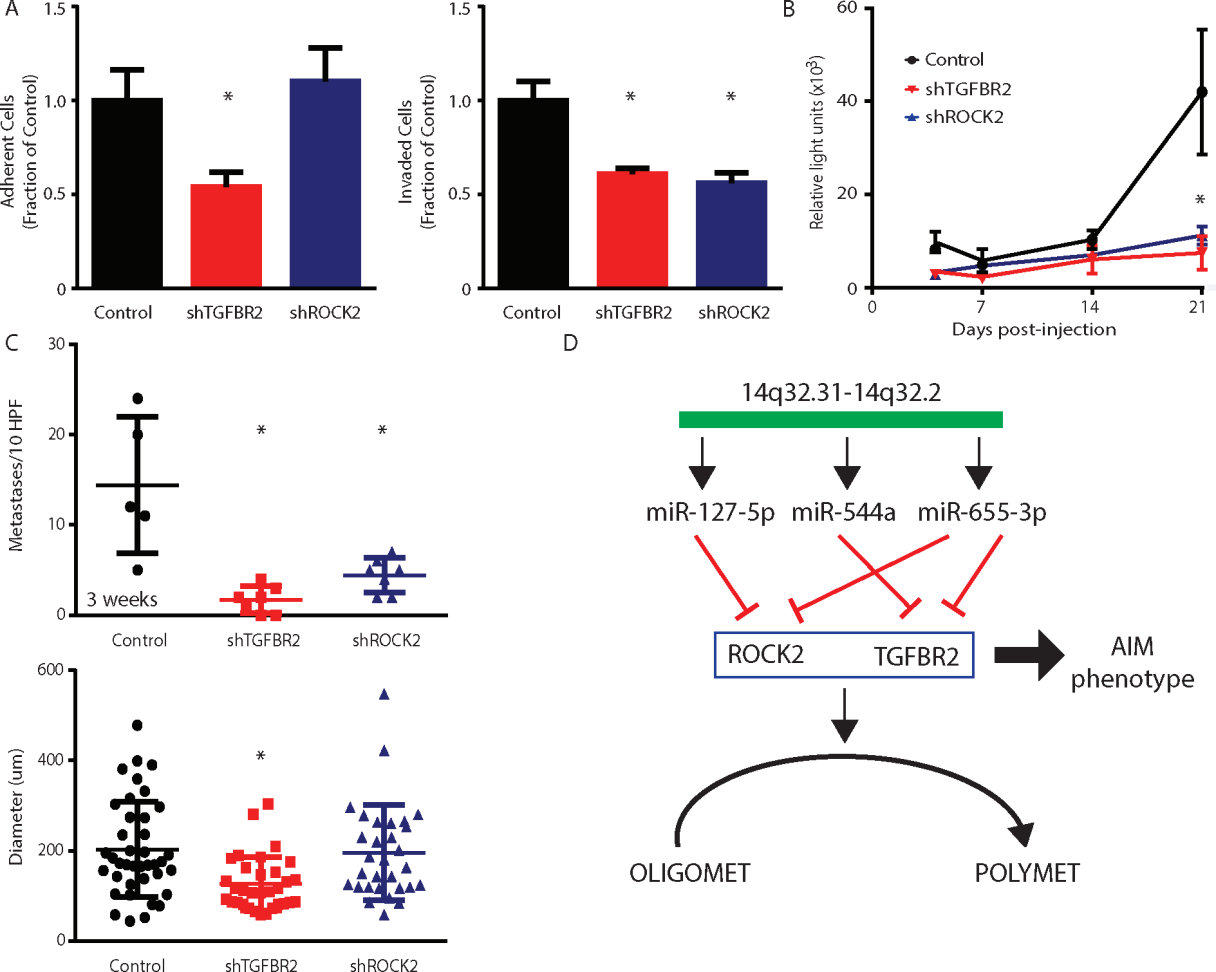
Stable suppression of microRNA target genes TGFBR2 and ROCK2 phenocopies oligometastatic state **(A)** Adhesion and invasion of MDA-MB-231 cells to Matrigel after stable suppression of *TGFBR2* or *ROCK2* using small hairpin RNA (shRNA) targeting each gene (*n* = 3 per group). **(B)** MDA-MB-231 polymetastatic breast cancer cells transfected with shTGFBR2, shROCK2, or a scrambled control and co-labeled with luciferase and GFP were injected into the tail veins of NOD/SCID mice. At 3 weeks, stable suppression of both genes led to a significant reduction in luciferase lung activity when compared to control. **(C)** Number and size of lung metastases per 10 HPFs at 3 weeks post-injection demonstrating significantly reduced numbers of lung metastases as a result of *TGFBR2* and *ROCK2* suppression (*n* = 5–7 per group). Micro-colony diameter at 3 weeks showed a growth delay due to *TGFBR2*, but not *ROCK2*, suppression when compared to control. Data represent mean ± SD. *p ≤ 0.05 compared to control. **(D)** Schematic of a proposed 14q32 microRNA-dependent regulation of an oligometastatic phenotype through co-repression of target genes.

We further examined whether knockdown of specific 14q32 miR target genes limited subsequent lung metastasis development. We used short hairpin RNAs (shRNA) against TGFBR2 and ROCK2 genes in the MDA-MB-231 cell line co-labeled with GFP and luciferase as was previously described. *In vitro* metastasis assays verified that *TGFBR2* knockdown significantly suppressed cellular adhesion and invasion (mean ± std. dev. relative to scrambled control; 0.54 ± 0.08, *p* = 0.004; 0.61 ± 0.03, *p* = 0.003; Student's *t*-Test; Figure 6A), while *ROCK2* knockdown significantly suppressed cellular invasion (0.56 ± 0.05, *p* = 0.04). *In vivo* lung colonization assays confirmed that both genes contributed to the metastatic competence of tumor cells in that suppression of either gene was sufficient to recapitulate a phenotype of limited metastases. At 3 weeks following tail vein injection, knockdown of *TGFBR2* or *ROCK2* significantly decreased (5.6-fold and 3.7-fold, *p* ≤ 0.05; Student's *t*-Test) the luminescence signal in the lungs of mice as compared to control treated animals (Figure 6B). These findings paralleled significant decreases in tumor micro-colony numbers for both *TGFBR2* (1.7 ± 1.5, *p* = 0.001) and ROCK2 (4.4 ± 1.9, *p* = 0.007) knockdowns when compared to controls (14.4 ± 7.6; Figure 6C). In addition, suppression of *TGFBR2*, but not *ROCK2*, led to a reduction in tumor micro-colony size at 3 weeks (Figure 6C). Taken together, these data suggested that either ectopic expression of selected 14q32-encoded miRs or stable repression of targeted genes by shRNAs leads to reversal of a polymetastatic phenotype to an oligometastatic phenotype (Figure 6D).

## DISCUSSION

We previously proposed oligometastasis as a potentially curable state existing between absent and widespread metastatic disease [1, 2]. In the current report, we present the first investigation into the biological basis for oligometastasis. We identified miRs associated with clinical oligometastasis in the only known datasets of patients with oligometastatic disease [4, 5]. Oligometastasis-associated miRs were predicted to suppress key metastatic pathways, including those mediating cellular adhesion, invasion and motility (AIM pathways) and multiple intracellular signaling pathways (such as TGF-beta, PI3K-Akt, ErbB, and mTOR). Gene expression profiling after ectopic expression of oligometastatic miRs validated co-regulation of these pathways by multiple oligometastatic miRs. These pathways are critically involved in phenotypic changes required during the multistep process of metastasis development [11, 12, 15]. Loss or deficiency in any component of these pathways could potentially reduce metastatic proficiency. These findings suggested that gene targets of oligometastatic miRs converge on several important metastatic pathways which might be critical in the development and/or maintenance of an oligometastatic state.

We also detected a significant enrichment of oligometastatic miRs encoded in the maternally imprinted DLK1-DIO3 region of chromosome 14, locus 14q32.2–14q32.31. Notably, miRs encoded in this locus have been implicated in oncogenesis and metastasis of diverse cancer types [8, 15]. In particular, multiple miRs within this locus have been implicated in the repression of EMT in tumor cells. Co-expression of miRs encoded in this locus suggests a common regulatory mechanism potentially through aberrant DNA methylation of the MEG3 region [16, 17, 24–27].

The molecular basis for co-regulation of these miRs may, at least in part, be related to the presence of binding sites for multiple miRs within the 3′ UTR of target genes. We identified several genes with multiple binding sites for oligometastasis-associated miRs. For instance, *TGFBR2* contains potential binding sites for miR-544a and miR-655-3p, *ROCK2* and *CDH11* contain binding sites for miR-127-5p, miR-655-3p and miR-369-3p and *ICK* contains sites for miR-544a, miR-655-3p and miR-369-3p. These genes were significantly suppressed by at least three of the four miRs based on gene expression profiling experiments and demonstrated similar patterns of suppression with 3′ UTR luciferase reporter assays.

Oligometastatic miRs co-repress target genes involved in the regulation of cellular adhesion and invasion properties of tumor cells. For instance, ROCK2 is a key mediator of the actin cytoskeleton that facilitates cell motility. Suppression of ROCK2 has been demonstrated to inhibit cellular invasion and metastasis formation [23]. In addition, small-molecule inhibitors of ROCK2 have demonstrated efficacy in preventing invasion of breast cancer cells [19]. CDH11 is a member of the cadherin family and is involved in cellular adhesion that is important for mediating bone metastases [22]. CDH11 over-expression is associated with poor recurrence-free survival in breast cancer, and depletion of its transcription factor HOXC8 resulted in fewer metastases in animal models [28]. The role of TGF-beta signaling is complex with the current understanding that it may inhibit growth in the early stages of tumor development, but promote invasiveness and growth in later stages that may progress to metastasis [15, 18]. ICK recently emerged as an oncogenic mediator in colon cancer [26] and an important regulator of the Hedgehog signaling pathway involved in tumor progression and metastasis [21]. Collectively, these data support a role for oligometastasis-associated miRs in complex regulatory networks inhibiting metastasis progression at several stages.

MiR-655 has previously been demonstrated to suppress TGFBR2 resulting in reduced invasiveness *in vitro* [30], which we confirmed in the context of an oligometastatic phenotype. In addition, we identified miR-544a as also targeting the TGF-beta pathway and capable of modulating metastasis development through inhibition of adhesion and invasion. This biological effect of limiting, but not fully inhibiting metastasis, is consistent with tumor plasticity and suggests that the behavior and evolution of metastatic tumors may be highly dependent on the pleiotropic effects of miRs [31].

The functional effects of these miRs on metastatic phenotypes *in vitro* and in a model of lung metastatic colonization supported the hypothesis that the early phases of metastatic colonization, involving adhesion and invasion into the host organ, play an important role in limiting metastasis number. miR-127-5p, miR-544a and miR-655-3p limited the number, but not size, of metastases *in vivo*. These three miRs also suppressed invasion *in vitro*. miR-369-3p did not significantly affect the development of lung metastases in this experimental model. Recent literature has identified alternative mechanisms of post-transcriptional regulation by miR-369-3p, which are potentially cell cycle- and cell type-dependent [32, 33]. In contrast, our findings suggest that miR-127-5p, miR-544a and miR-655-3p might promote a similar oligometastatic phenotype through suppression of adhesion and invasion pathways. In addition, these results suggest that proliferation of established micro-colonies may play a relatively limited role in the early development of oligometastasis. Importantly, the results demonstrate that individual miRs can mediate the development of oligometastasis by modifying the equilibrium between pro- and anti-metastatic effects, which may drive an intermediate phenotype such as oligometastasis.

This study begins with a clinical observation that metastases can be limited in number and location, and thus are amenable to localized treatments. The results presented in this report provide support to the concept of metastasis as a spectrum of diseases with varying complex phenotypes. Notably, our results differ from previous studies of differential miR expression between non-metastatic and widely metastatic states, or between primary and metastatic tissue within the same subject, because our findings identify characteristics of an intermediate metastatic phenotype and reveal the significant molecular heterogeneity of metastatic lesions. These results support our hypothesis of oligometastasis as a clinical entity with biological mechanisms and molecular properties that may differ from polymetastatic disease. Collectively, these findings suggest the existence of multiple pathways that can modify the essential capacities necessary for metastasis development. These data suggest that multiple miRs can produce a similar metastatic phenotype due to their convergent actions on target genes governing metastatic potential. Further phenotypic studies of miRs derived from oligometastasis, and their target genes, may lead to an improved understanding of the processes that modulate metastatic progression. Our results set the stage for improved identification of patients with oligometastasis and guide the development of therapies to limit metastasis development.

## METHODS

### MicroRNA target pathway prediction

Patient-derived samples of surgically resected lung metastases (surgical dataset) and stereotactic body radiotherapy-treated metastases from various organ sites (SBRT dataset) were classified as oligometastatic or polymetastatic and analyzed using TaqMan MicroRNA Array A Card v2.0 as previously described [4, 5]. These data are available at the Gene Expression Omnibus (GSE25552 and GSE38698). Predicted targets of each microRNA were determined using Diana microT-CDS (MicroT threshold 0.6) [34]. KEGG pathways were identified using hypergeometric testing via Diana miRPath v2 (*p* ≤ 0.05). Statistical significance of each KEGG pathway was calculated using Fisher's combined probability method via Diana miRPath v2 [35].

### Kaplan-Meier survival analysis

Kaplan-Meier curves for recurrence-free survival were constructed by grouping patients into high-expressing (average microRNA expression of miR-127-5p, miR-369-3p, miR-544a and miR-655-3p greater than the group median) and low-expressing cohorts. Statistical significance was determined using a log-rank test.

### MicroRNA genomic distribution analysis

Genomic locations of microRNAs were examined and statistical significance of overlapping loci was determined with random permutation testing. Frequencies of specific loci were compared between differentially expressed microRNAs and all microRNAs present on the array. Statistical significance was determined by randomly choosing a particular microRNA from the differentially expressed set or the entire array and comparing the incidence of belonging to a particular genomic locus. This was repeated 10^6 times to obtain a null distribution, as well as to compare the incidence of 14q32-encoded microRNAs to all microRNAs present on the array.

### Gene expression profiling and analysis

MDA-MB-231 cells transfected with selected microRNAs were collected in cell lysis buffer, and RNA was isolated using the RecoverAll Total Nucleic Acid Isolation kit (Ambion). 100 ng of RNA was labeled per manufacturer's instructions and profiled in duplicate using the Illumina Human HT12v4 array (Illumina, San Diego CA). Background subtraction and quantile normalization was performed across arrays using Illumina Beadstudio software. Log-transformed gene expression was compared using Significance Analysis of Microarrays (SAM) for Excel (Stanford University, CA) with a False Discovery Rate (FDR) of 5% and a fold-change threshold of greater than or equal to 1.4 to identify differentially expressed genes [36].

Overlapping gene probesets by individual microRNAs was illustrated using the Vennerable package in R. Significance of overlap was determined using Chi-squared contingency tables calculated in R by applying a population size corresponding to the total number of probes detected on all four arrays for each microRNA pair examined.

Ingenuity Pathway Analysis (IPA, Redwood City CA) was used to identify over-represented canonical pathways. A fold-change threshold greater than or equal to 2.0 was applied to gene sets prior to analyses. Over-representation of gene sets in a canonical pathway was calculated using hypergeometric testing with an alpha value of 0.05. Significantly enriched pathways were manually distributed into specific functional groups. Cytoscape 3.0.2 was used to identify pathways enriched by multiple microRNA target gene sets.

### Cell lines and culture

The Luc2-GFP plasmid and MDA-MB-231 cell line were obtained as a gift from Dr. Geoffrey Greene at the University of Chicago. The cell line was maintained in DMEM with 10% fetal bovine serum and 1% penicillin/streptomycin.

### MicroRNA transfection

MDA-MB-231 cells were forward transfected with Dharmafect Reagent 1 and microRNA mimics (Dharmacon, Pittsburgh PA) at a concentration of 50 nM per the manufacturer's instructions. Non-targeting mimics labeled with Dy546 fluorescence epitope were used as negative controls and to determine transfection efficiency. Transfection medium was replaced after 24 hours. Cells were harvested at 48 hours post-transfection. The percentage of living cells within each group was determined using Tryptan Blue staining. Transfection efficiency and cell survival greater than 90% was routinely achieved.

### Short hairpin RNA gene knockdown

MDA-MB-231 cells were transduced with lentivirus encoding either scrambled shRNA or a shRNA targeting TGFBR2 or ROCK2 mRNA for knockdown. Lentiviral vectors were obtained from Promega (TGFBR2: TRCN0000040011; ROCK2: TRCN0000196480). Monolayers of 293T cells were co-transfected with plasmid encoding surface glycoprotein VSV-G, lentiviral structural proteins and shRNA-containing lentiviral genome. At 48 hours after transfection, cell supernatant was collected and used to transduce MDA-MB-231 cells at an approximate MOI of 0.01. Transduction was performed in the presence of polybrene (8 μg/ml). Media was replaced after 24 hours. Puromycin (2 μg/mL) was added 3 days after transduction to media to select for stably transduced cells expressing puromycin resistance gene from the lentiviral genome. After 3 days of selection, colonies of resistant cells were isolated and expanded into cell lines used for subsequent experiments. Thereafter, cells were maintained in puromycin-containing media. Efficiency of protein depletion was determined by Western blot using the following antibodies: TGFBR2 (sc-17799), ROCK2 (sc-5566) and β-actin (sc-47778) (Santa Cruz) (Supplemental Figure S3).

### Adhesion assay

MDA-MB-231 cells were harvested 48 hours after microRNA transfection and plated at a density of 20,000 cells per well in 96-well plates pre-coated with Matrigel (BD Biosciences). Five replicates were performed per treatment group. After 2 hours of incubation, cells were gently washed twice with media. Adherent cells were incubated with CellTiter-Blue for 3 hours and relative fluorescence units quantified after 3 hours per the manufacturer's instructions. Signals were normalized to non-targeting control.

### Invasion assay

MDA-MB-231 cells were harvested 48 hours after microRNA transfection and plated at a density of 100,000 cells per well in DMEM into 24-well 8 μm pore transwell inserts pre-coated with Matrigel (BD Biosciences) per the manufacturer's instructions. Three replicates were performed per group. Bottom wells were loaded with DMEM containing 10% FBS. After 48 hours, cells were removed from the top of the transwell membrane, and the membranes were stained with DAPI 1:1000 in PBST. Migrated cells were counted for 5 high-powered fields per well, averaged and normalized to control.

### Lung metastatic colonization model

MDA-MB-231-L2G cells (3 × 10^6^) were transfected with 50 nM of microRNA or mimic as described above. After 48 hours, cells were labeled with VivoTrack 680 (Perkin-Elmer) and re-suspended in cold PBS. Six NOD.SCID mice (Jackson Labs) were injected with 5 × 10^5^ cells per 100 uL of PBS via tail vein. Mice were imaged at 5, 7, 14, and 21 days using the IVIS 200 (Xenogen) imaging system after intra-peritoneal injection of 150 μg of luciferin. Data were analyzed using LivingImage 4.0 Software (Caliper Life Sciences) and expressed as total photon flux. This was repeated for a second cohort of 9 mice per group, and luciferase data were combined for statistical analysis by normalizing to the non-targeting control of each cohort at each time point. Lungs were imaged with the Leica DP5 confocal microscope to quantify colonies per animal and metastasis diameter. Ten fields at 20× magnification were obtained per animal and the number of metastases within these fields was determined. Whole lung images were obtained with an Axiovert 200c Inverted Microscope.

### 3′ UTR luciferase reporter assay

Twenty four hours prior to transfection, HEK 293T cells were plated in a 96-well plate to a density of 15,000 cells per well. Cells were subject to two sequential transfections: (1) microRNA mimics or a non-targeting control (50 nM) and after 24 hours (2) *Renilla* luciferase vector (pLightSwitch_3UTR, 100 ng per well, Switchgear Genomics). Twenty-four hours after the second transfection, monolayers were frozen at −80°C, lysed and reacted with luciferase reagent (Switchgear Genomics) according to the manufacturer's recommendation. Luciferase activity was quantified over 2 seconds per well using a Tecan Safire 2 plate reader. Values represent percent activity relative to control-treated cells. All experiments were performed in triplicate. Transfection with *Renilla* luciferase vector (pLightSwitch_3UTR with no predicted binding sites) was performed as a negative control, and no suppression of signal was noted for microRNAs compared to non-targeting control.

### Site-directed mutagenesis

Plasmids encoding mutated microRNA binding sites were generated using the Stratagene Quick Change site directed mutagenesis kit (Stratagene, La Jolla, CA). Briefly, plasmid DNA encoding the wild-type 3′ UTR sequence was isolated from methylation competent *E. coli* and amplified by PCR with primers encoding nucleotide substitutions predicted to abrogate microRNA binding. Primer sequences are available in Supplemental Figure S3. Parental plasmid was removed by digestion with methylation-specific restriction enzyme DpnI. The remaining DNA was amplified in *E. coli* and purified using standard procedures. All mutations were confirmed by sequencing.

## SUPPLEMENTARY FIGURES AND TABLES




