# Supplementary material for: 14q32-encoded microRNAs mediate an oligometastatic phenotype
Source: Oncotarget. 2015 Feb 18;6(6):3540–52. doi: 10.18632/oncotarget.2920 (PMC4414135; doi:10.18632/oncotarget.2920)
Supplement: Supplementary file 1 [file oncotarget-06-3540-s001.pdf]

SUPPLEMENTARY FIGURES AND TABLES

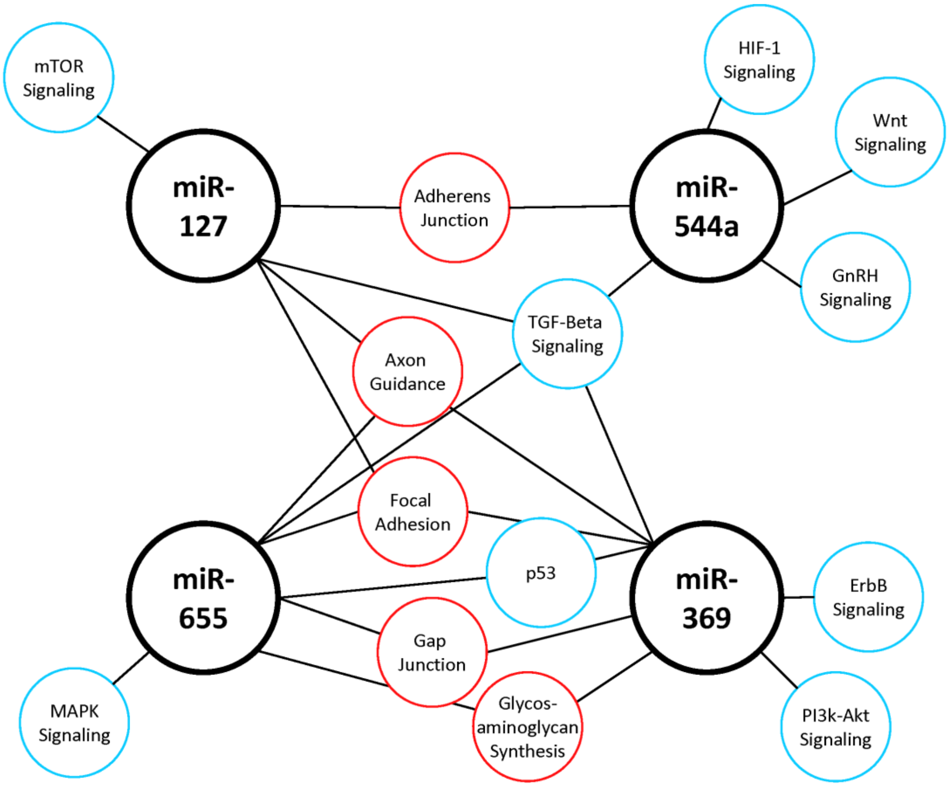

Supplemental Figure S1: Oligometastatic microRNAs comprise central nodes in a gene network connecting adhesion, invasion, and motility phenotypes (red circles) to known metastatic signaling pathways (blue circles).

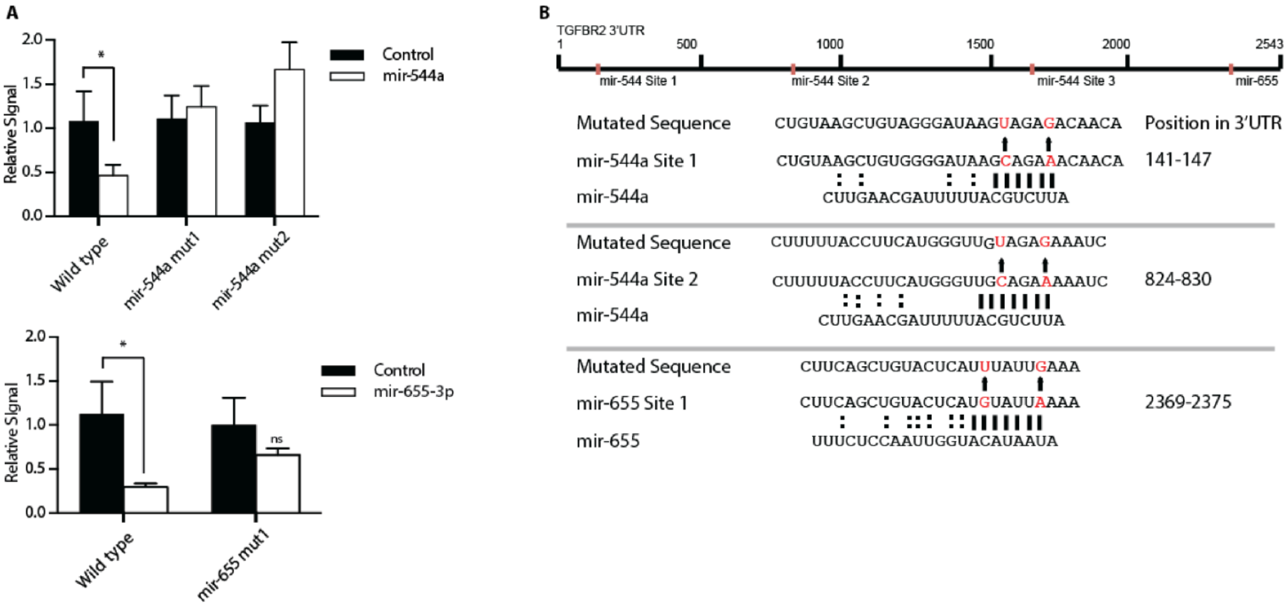

Supplemental Figure S2: Mutagenesis of predicted microRNA binding sites within the 3' untranslated region (UTR) of *TGFBR2* in HEK 293T cells. (A) TGFBR2 3' UTR luciferase reporter assay after site-directed mutagenesis of miR-544a (top) and miR-655-3p (bottom) binding sites demonstrating an abrogation in luciferase signal reduction as a result of binding site mutagenesis. $*p \leq 0.05$. **(B)** Schematic of mutations in *TGFBR2* 3' UTR created by site-directed mutagenesis. Two binding sites for miR-544a and the single binding site for miR-655-3p were each mutated with two point mutations (illustrated in red).

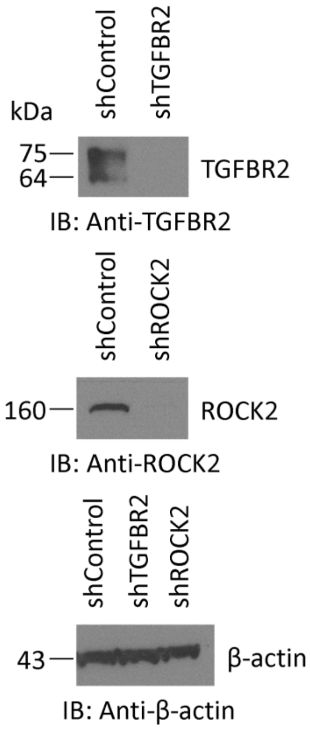

Supplemental Figure S3: Western blot confirmation of gene knockdown in MDA-MD-231 cells transduced with a lentivirus encoding either scrambled shRNA (shControl) or shRNA targeting TGFBR2 (shTGFBR2) or ROCK2 (shROCK2) mRNA for knockdown. IB: Antibody used for immunoblot. β-actin served as a loading control.

Supplemental Table S1: Numbers of predicted target genes for each miR over-expressed in patient-derived samples of surgically resected lung oligometastases (surgical dataset) and stereotactic body radiotherapy-treated oligometastases from various organs (SBRT dataset)

Surgical miRNA	# predicted targets
128	1045
153	600
298	361
328	135
329	790
412	321
448	658
655	829
887	10
127-3p	15
127-5p	290
133a	557
135a-5p	644
154-5p	74
191-5p	25
199a-5p	302
199b-5p	303
296-3p	104
299-3p	253
323b-5p	104
330-5p	685
369-3p	1195
380-3p	650
485-3p	204
485-5p	360
491-5p	296
502-5p	332
520a-3p	703
520g	173
541-3p	454
544a	796
576-5p	569
582-5p	830
let-7b-5p	638
let-7c	616

SBRT miRNA	# predicted targets
328	135
489	82
125a-3p	52
140-3p	386
140-5p	279
193a-3p	225
199b-5p	303
29c-3p	585
331-5p	32
363-3p	419
500a-5p	403
502-5p	332
545-3p	218

Supplemental Table S2: KEGG pathway enrichment for each microRNA over-expressed in oligometastases from the surgical dataset

Supplemental Table S3: KEGG pathway enrichment for each microRNA over-expressed in oligometastases from the SBRT dataset

KEGG Pathway	miR-500a-5p	miR-328	miR-125a-3p	miR-140-5p	miR-29c-3p	miR-140-3p	miR-489	miR-331-5p	miR-193a-3p	miR-199b-5p	miR-502-5p	miR-545-3p	miR-363-3p
**Adhesion, Motility and Cytoskeleton (AIM)**													
Axon guidance (hsa04360)						3.1			4.2		1.4		
Focal adhesion (hsa04510)		2.0		1.8	16.6	1.6	1.6	1.7	3.7		1.7	4.0	2.3
Regulation of actin cytoskeleton(hsa04810)		2.2		2.1	1.6			3.2	2.2				3.6
ECM-receptor interaction (hsa04512)					69.5								
Gap junction (hsa04540)	1.4		1.5				1.7	6.3			4.5	2.4	1.5
Amoebiasis (hsa05146)		2.4			19.3						2.1		
Glycosaminoglycan biosynthesis - chondroitin sulfate (hsa00532)					3.1						11.1		
**Intracellular Signaling (ICS)**													
TGF-beta signaling pathway (hsa04350)			1.8								4.0		
MAPK signaling pathway (hsa04010)		2.8						2.4	2.4			1.3	
PI3K-Akt signaling pathway (hsa04151)	2.3	2.2		1.6	10.0	3.3	1.4	2.6	2.4		2.3		6.3
ErbB signaling pathway (hsa04012)	1.4		1.5			2.4	2.1		2.5		6.2	1.8	1.5
Wnt signaling pathway (hsa04310)					2.6	2.1			4.2				

(*Continued*)

p53 signaling pathway (hsa04115)					3.1				3.7				2.1
mTOR signaling pathway (hsa04150)	1.5	2.4				1.8					1.5		2.4
Insulin signaling pathway (hsa04910)						3.6		1.4				1.7	1.4
Ubiquitin mediated proteolysis(hsa04120)	3.8											3.6	1.5
GnRH signaling pathway (hsa04912)									3.5			1.8	1.4
Phosphatidylinositol signaling system(hsa04070)	3.7										1.8		3.0
VEGF signaling pathway (hsa04370)	1.4				1.9								1.6
Fc epsilon RI signaling pathway(hsa04664)					2.1						1.7		
**Cancer-Type Specific Pathways (CSS)**													
Prostate cancer (hsa05215)			1.7		4.2		2.2	3.2	2.4	5.3	2.0		11.1
Pathways in cancer (hsa05200)					5.3			2.3	3.8				1.5
Endometrial cancer (hsa05213)							1.4	1.9	4.8		3.0		2.4
Colorectal cancer (hsa05210)				2.0	1.5				3.4		5.3		1.3
Small cell lung cancer (hsa05222)	1.5				15.6	3.0			3.2	2.1			4.5
Non-small cell lung cancer (hsa05223)			2.5		2.5	2.0			1.4		3.0		2.6
Pancreatic cancer (hsa05212)				1.8	3.1	1.5					3.3		2.9
Renal cell carcinoma (hsa05211)					2.8	2.1						1.7	1.3
Glioma (hsa05214)			2.7		3.4				2.8		2.2	2.6	2.3
Chronic myeloid leukemia (hsa05220)				1.8		2.3	1.6	2.1			4.9		3.0
Acute myeloid leukemia (hsa05221)							2.9	1.7	1.6		1.4	1.4	1.5
Melanoma (hsa05218)					3.6		1.4	1.7	2.3		2.3		2.6

Values indicate –log10 of *p*-value of hypergeometric test for each miRNA/KEGG pathway interaction.

Supplemental Table S4: Observed and expected numbers of co-repressed probesets between combinations of 14q32-encoded microRNAs in MDA-MD-231 cells

Combination	Predicted	Observed	Observed/ Predicted	*p*-value (Chi-square with Yates' correction)
127 – 369	200	344	1.72	< 0.0001
127 – 544	342	515	1.51	< 0.0001
127 – 655	278	519	1.87	< 0.0001
369 – 544	222	401	1.81	< 0.0001
369 – 655	179	767	4.29	< 0.0001
544 – 655	307	547	1.78	< 0.0001
127 – 544 – 655	40	165	4.12	
127 – 544 – 369	29	108	3.78	
127 – 655 – 369	23	68	2.9	
544 – 655 – 369	25	261	10.3	
127 – 369 – 544 – 655	3	76	22.2	

Differentially expressed genes were identified using a fold-change threshold of 1.4 and false discovery rate of 5%.
